# Integrated laboratory information system in a large hospital laboratory in Singapore

**DOI:** 10.1155/S1463924692000403

**Published:** 1992

**Authors:** Edward Jacob, It-Koon Tan, Kim-Seng Chua, See-Heng Lim

**Affiliations:** Clinical Biochemistry Laboratories Department of Pathology Singapore General Hospital Outram Road Singapore 0316 Singapore

## Abstract

This paper describes an integrated approach to the computerization of all major disciplines of laboratory medicine and pathology. Installed in the Department of Pathology, Singapore General Hospital (SGH), the computer system discussed comprises a RISC-based Data General Aviion 6200 computer and Meditech MAGIC software. The system has been interfaced with the hospital host IBM computer and supports patient information transfer, result reporting, phlebotomy management, and compilation of laboratory and financial management reports. The main functions of the system include: on-line and off-line acquisition of patient information and test data; preparation of single/combined/cumulative reports; transmission of reports within and between laboratories; instantaneous provision of data in response to telephone enquiries; calculations of quality control/workload/productivity statistics and indices; and generation of billing lists. The computer enables reports to be provided on patient tests results in individual wards, at various specialist out-patient clinics, and in the Accident and Emergency Department of the SGH through the IBM mainframe, as well as to remote printers installed at several other major hospitals.

The use of the MAGIC integrated laboratory information system has resulted in a significant increase in laboratory efficiency and productivity.


Journal of Automatic Chemistry, Vol. 14, No. 6 (November–December 1992), pp. 223–229

### Integrated laboratory information system in a large hospital laboratory in Singapore

**Edward Jacob, It-Koon Tan, Kim-Seng Chua and See-Heng Lim**

*Clinical Biochemistry Laboratories, Department of Pathology, Singapore General Hospital, Outram Road, Singapore 0316, Singapore*

*This paper describes an integrated approach to the computerization of all major disciplines of laboratory medicine and pathology. Installed in the Department of Pathology, Singapore General Hospital (SGH), the computer system discussed comprises a RISC-based Data General Aviion 6200 computer and Meditech MAGIC software. The system has been interfaced with the hospital host IBM computer and supports patient information transfer, result reporting, phlebotomy management, and compilation of laboratory and financial management reports. The main functions of the system include: on-line and off-line acquisition of patient information and test data; preparation of single/combined/cumulative reports; transmission of reports within and between laboratories; instantaneous provision of data in response to telephone enquiries; calculations of quality control/workload/productivity statistics and indices; and generation of billing lists. The computer enables reports to be provided on patient tests results in individual wards, at various specialist out-patient clinics, and in the Accident and Emergency Department of the SGH through the IBM mainframe, as well as to remote printers installed at several other major hospitals.*

*The use of the MAGIC integrated laboratory information system has resulted in a significant increase in laboratory efficiency and productivity.*

### Introduction

The Department of Pathology of the Singapore General Hospital (SGH) provides both routine and specialized laboratory services to hospitals and clinics, and to government ministries. The Department acts as the reference and training centre for laboratory medicine in Singapore and the region.

An off-line minicomputer system was installed in the Biochemistry Division of the Department of Pathology in 1974; the workload was then 426 000 tests per annum. The off-line system, which comprised a NOVA 1220 computer with 48KB memory, paper tape reader, several teletypes and printers, served the laboratories well for about 10 years [1–5].

With a doubling of the workload to about 820 000 tests per annum in 1980, and the aging of the minicomputer, it was felt that a new computer was needed. An exhaustive study of laboratory computers on the market, and the experiences of other hospital laboratory computer users [6–17], led to the final choice of an interactive multi-user database management operation system. The computer was a Data General Eclipse S/140 minicomputer running on Medical Information technology (Meditech) Software written in Meditech Interpretive Information System (MIIS). Two identical on-line systems were purchased, one in 1983 to serve the haematology laboratories and the 24-hour emergency/routine biochemistry laboratories sited on the same floor within SGH, and another in 1984, to serve the main biochemistry laboratories of the Department of Pathology which is housed in a separate building some distance away. The other laboratories at SGH were not ready for computerization.

The annual number of tests in the biochemistry and haematology departments was 820 000 and 700 000, respectively, when the two computer systems were purchased. The systems were linked through modems and telephone lines and have performed well [18].

With a background of many years of laboratory computing experience in the field of biochemistry and haematology, it was decided in early 1990 to computerize all the divisions in the Department of Pathology. It was decided that a single large computer system could cater for all of the divisions. This was considered to be a better approach than using small individual systems for each separate discipline—for example the various divisions of the Department would be able to share the same patient database (see table 1).

### Configuration of the newly-acquired MAGIC system

The group had many years' experience with staff with the Meditech software and it was felt that this outweighed any small advantages that any other system could offer and decided on an upgraded design from Meditech, Inc. called the MAGIC System.

The system consists of a Data General RISC based AVIION 6200 Series Computer with 16 MB memory,

*Table 1. Number of specimens processed in 1990.*

| Division | No. of specimens |
|---|---|
| Biochemistry | 443 132 |
| Haematology | 275 158 |
| Diagnostic Bacteriology | 224 783 |
| Microbiology | 100 351 |
| Serology/Immunology | 213 295 |
| Virology | 74 845 |
| Histopathology | 50 743 |
| Cytology | 59 100 |
| Total | 1 441 407 |

0142–0453/92 $10.00 © 1992 Taylor & Francis Ltd.

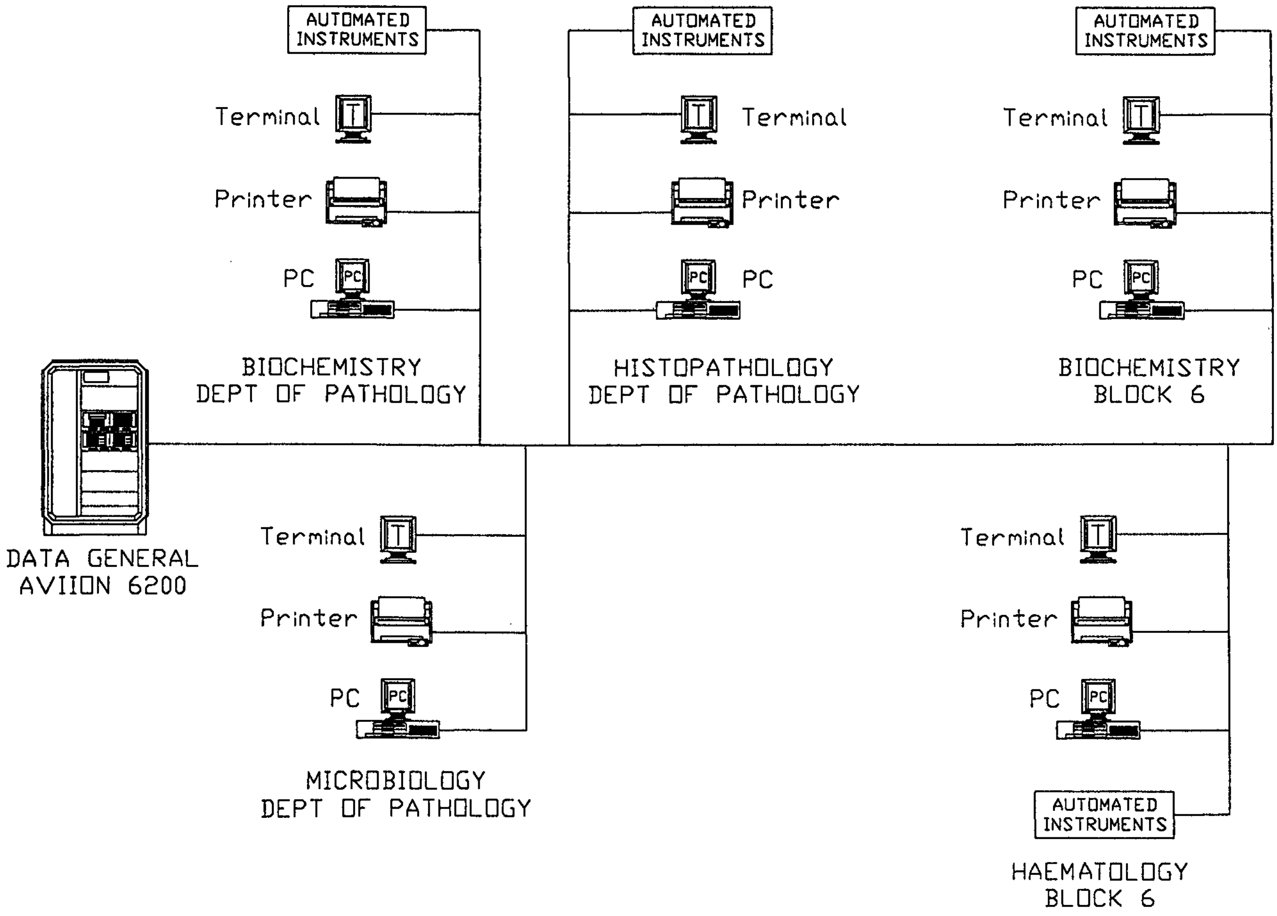

*Figure 1. Layout of the integrated laboratory computer system in the Department of Pathology, Singapore General Hospital (SGH).*

3 GB disc storage, 2·3 GB helical tape drive, Ethernet interface, 24 units of communication servers providing 10 serial ports each connected to the Ethernet Local Area Network. The 3 GB disc storage is made up of three units of 1 GB disc drive. It is organized with one unit as the master, one unit as the shadow and one for archiving. The system layout is shown in figure 1.

The CPU, the console VDU, the live and archival disc drives and the helical tape drive are housed in the Computer room of the Department. Cables on an Ethernet network link terminals from the various laboratories to the CPU.

### Networking

As the laboratories of the various disciplines are distributed over a wide area within the department, it was necessary to connect all the peripheral devices in each laboratory to the central CPU through a cable network. A cable system to link all the peripherals within the hospital was adopted as it allowed higher speed of communication (19·2 baud rate) and minimal incremental cost for additional links in the future (see figure 2). To serve the hospital's future needs a fibre-optic cable was laid in the underground tunnel of the hospital (see figure 3).

The specifications of the fibre-optic cable are: four core, multimode and 62·5/125. It is, however, running at Ethernet bandwidth of 10 MBit/s; it will be upgraded to a Fibre Distributed Data Interface (FDDI), with a speed of 100 MBit/s, in the future.

### Software

The operating system is the MAGIC Operating System, designed by Meditech. The computer language is the MAGIC language and the application programme modules which are in use are as follows:

(1) Laboratory—for biochemistry, haematology and nuclear medicine.

(2) Microbiology—for diagnostic and enteric bacteriology, serology, immunology and virology.

(3) Anatomical pathology—for histopathology and cytopathology.

(4) Accounts receivable—for finance and billing.

The software is an integrated package running on a common database.

### Features of the MAGIC computer operations

Each section of the Department uses the computer independently and is responsible for its own patient data input, request input, workload production, result entry,

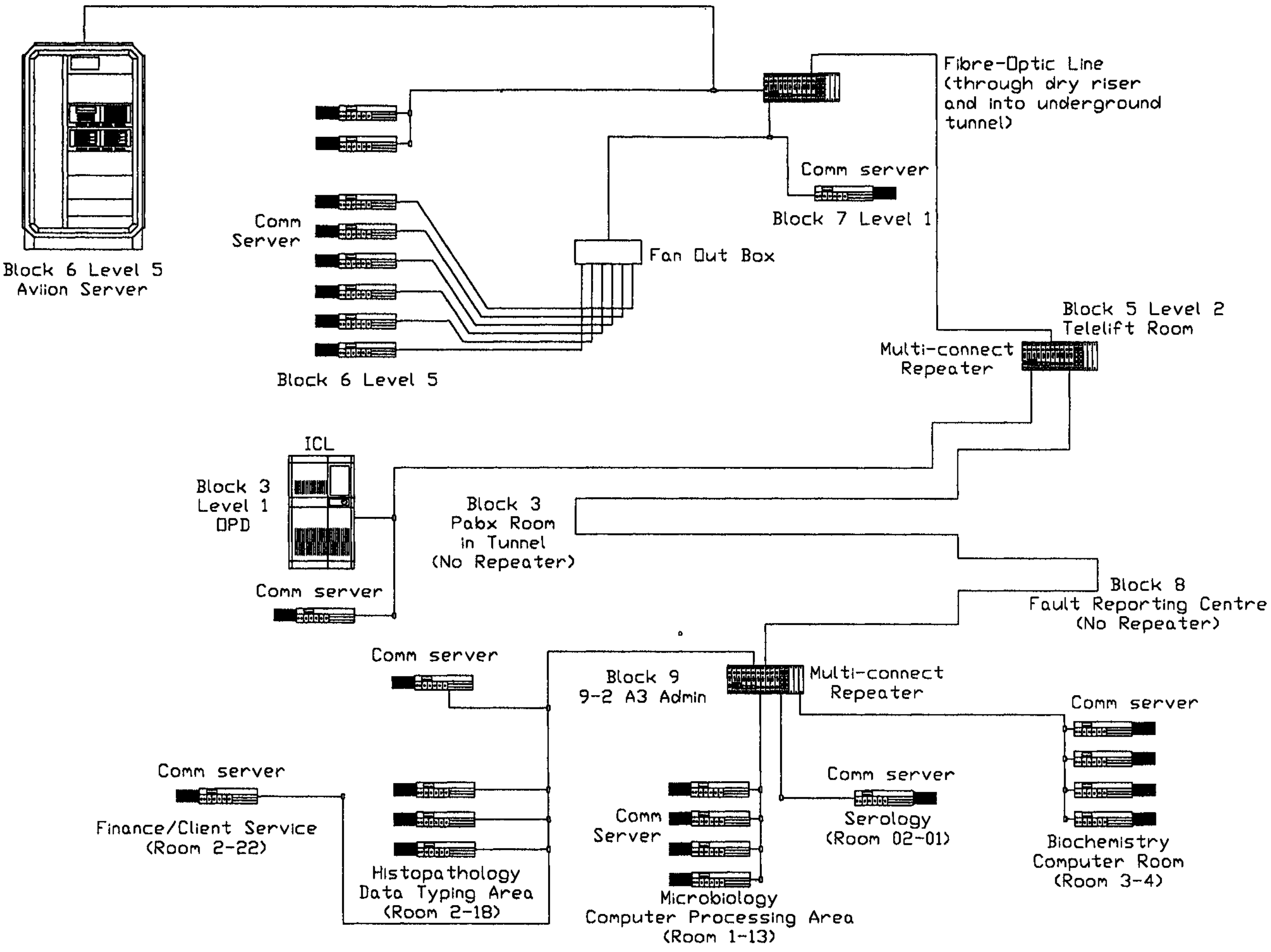

*Figure 2. Cable system linking all the computer peripherals in the various buildings of the hospital.*

report printing, record purging and archiving, statistical analyses and workload production. The various laboratory disciplines have been alloted their own independent disc space in the live and archival disc. Access to each group's disc space is by separate passwords.

The integrated package provides a common database for demographic data. This has resulted in a considerable saving in manpower in terms of data entry: once patient is admitted into the system, this information can be accessed by any of the laboratory users.

Some customization had to be done to suit local requirements: such additional fields as requisition numbers, race, financial class and service codes have been added. New programs were written for workload statistics for accumulating the number of tests done in each laboratory.

*Transfer of demographic data*

Patients admitted to SGH have their demographic data keyed into the Hospital's host IBM computer Patient Care System (PCS). This information is downloaded on a real-time basis to a personal computer (PC) sited in the laboratory. The data are processed and then uploaded to the Meditech's patient database. Ward transfers, bed changes, discharges are entered at the nursing stations into the host IBM computer. These transactions are automatically passed to the laboratory PC, which then updates the Meditech's patient database (see figure 4). This is an excellent feature because all laboratories have real-time updated patient demographic data; ward enquiries and results reporting are now faster and more efficient.

*Meditech to IBM result reporting*

The laboratory system is linked to the Hospital IBM 3090 System for demographic transfer—the link is through a PC. All test results from the laboratory system, after verification, are transmitted via a microcomputer through a protocol converter to the IBM host.

The results are initially downloaded to the PC and a hard copy is printed for verification. Unsatisfactory results can be suppressed at this stage and the rest transmitted to the host computer. The IBM computer then transmits all results to IBM printers located in the wards (see figure 5). This has improved the turnaround time for result reporting. Reports are generated throughout the day, and ward staff have been given the facility of initiating a printout of reports through the ward terminals.

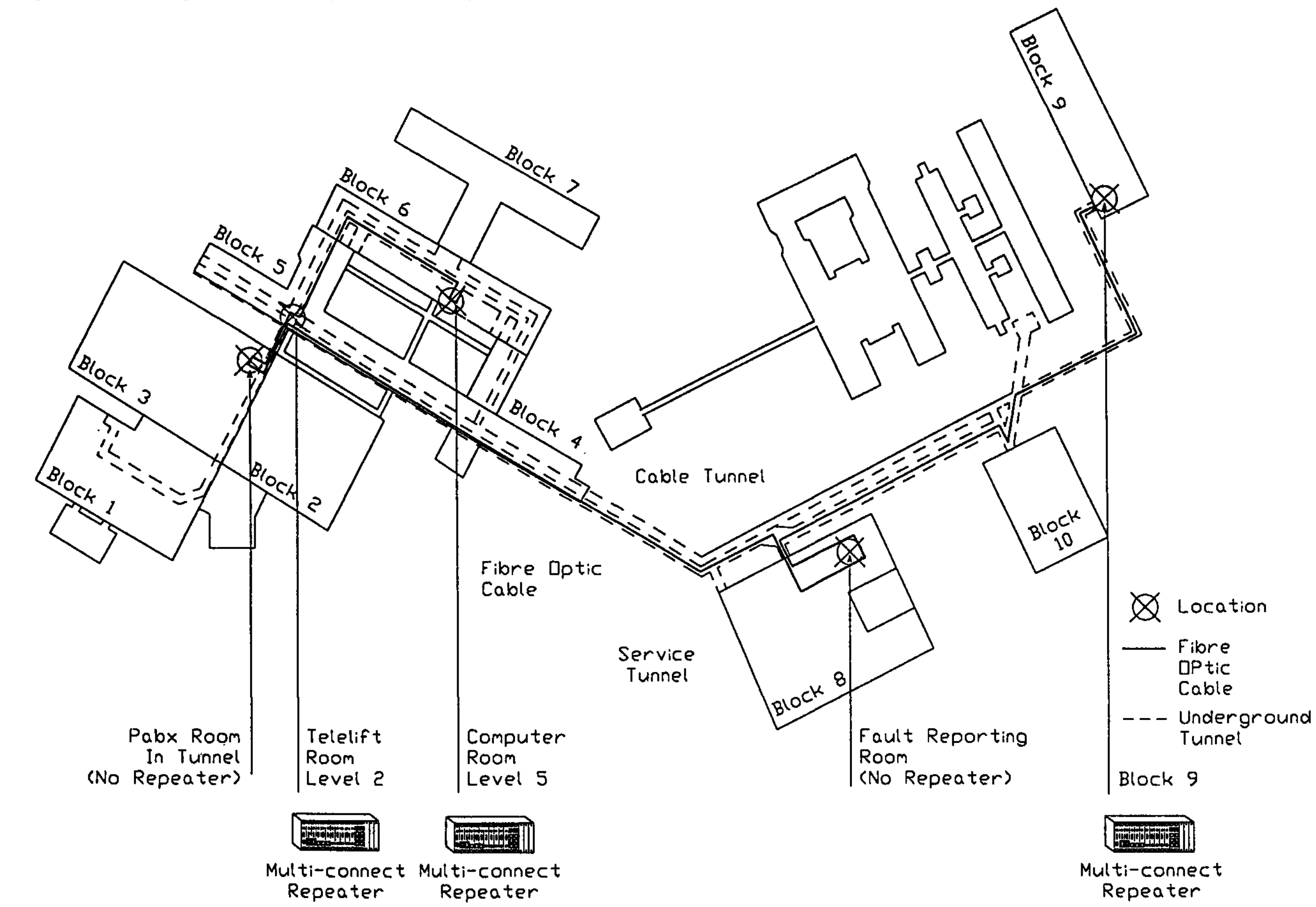

*Figure 3. Fibre-optic cable network laid in the underground tunnel of the hospital.*

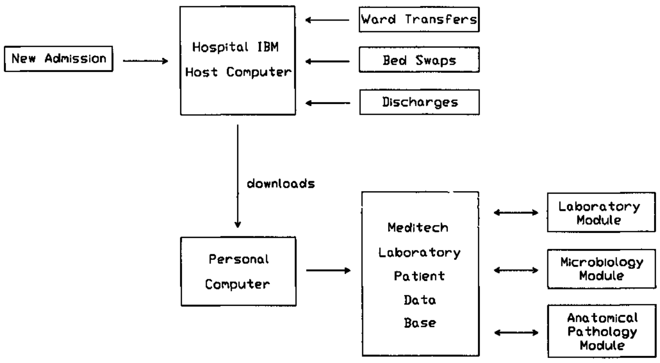

*Figure 4. Automatic transfer of patient demographic information from the hospital mainframe computer to the laboratory information system through a personal computer.*

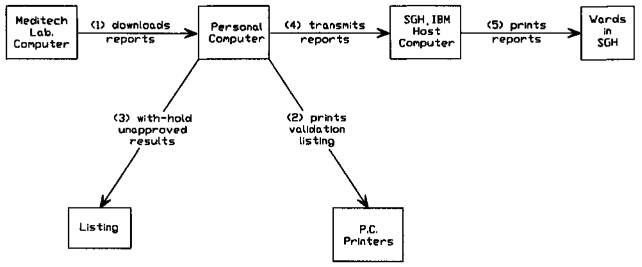

*Figure 5. Transmission of laboratory test results from the laboratory information to the hospital mainframe computer for the printing of reports within the wards and out-patient departments.*

*Report formats*

The MAGIC software allows for composite reports featuring results for a patient from all laboratory disciplines. The present arrangement, however, is for each discipline to generate their own separate reports.

There are a number of options for the format of reporting by the system for the various laboratory disciplines. Figures 6–9 are examples of single reports generated by the system for some of the laboratories. The histopathology division was one of the last to be computerized but it has made considerable gains. A large part of histology reporting is in text form and requires word processing; this is done efficiently through the MAGIC software. The system uses a laser printer and reports are neat and well presented. A major bonus is automatic SNOMED coding of diagnosis. Each division of the Department selects its reports based on prefixes created. Biochemistry and haematology reports carry age-specific reference intervals and delta checks and panic values are used to highlight, or flag, abnormal results. Free text is allowed in the comment field to describe, for example, haemolysis, turbidity or to alert the clinician that the specimen was inappropriate and a repeat specimen was desirable.

*Result reporting to outside clients*

Laboratory reports are transmitted via telephone lines through modems to be printed at remote printers located on site in other hospitals and the premises of clients who are important users of the Department's services (see

Singapore General Hospital

Department of Pathology: Telex: RS 28829 PATLAB Fax: 2228924

RUN ON 16/09/91-1500 — Singapore General Hospital BIOCHEMISTRY REPORT — PAGE 1 RUN FOR 16/09/91

A,B C — SGH WARD 48 — NRIC # U1234567

MALE AGE: 59 — CLASS: A RM/BED: — ID # C162

1609:BC0001S REQUEST # BC543276 RECV: 16/09/91 UNK COLLN: 16/09/91 UNK

ORDERED: UE,CRE,GLU, (CALCIUM,PO4), LFT/ALT/AST, LDH, CHO,TG,HDL,LDL, PROSTATE SP AG, ICD, CEA, (T3U,T4,FTI), ALPHAFOETO PROT

| | | | |
|---|---|---|---|
| [UE,CRE,GLU] | | | |
| UREA | 5.6 | MMOL/L | (2.8-7.7) |
| SODIUM | 142# | MMOL/L | (135-145) |
| POTASSIUM | 4.9# | MMOL/L | (3.3-4.9) |
| CHLORIDE | 106# | MMOL/L | (96-108) |
| GLUCOSE | 9.0# | MMOL/L | (3.1-10.0) |
| | | FASTING | (3.1-6.6) |
| CREATININE | 91# | UMOL/L | (44-141) |
| [(CALCIUM,PO4)] | | | |
| CALCIUM,TOTAL | 2.27 | MMOL/L | (2.10-2.60) |
| PHOSPHATE,INORG | 1.03 | MMOL/L | (0.77-1.38) |
| [LFT/ALT/AST] | | | |
| PROTEIN,TOTAL | 65# | G/L | (62-82) |
| ALBUMIN | 38# | G/L | (37-51) |
| BILIRUBIN,TOTAL | 18# | UMOL/L | (3-24) |
| ALKALINE PHOSPH | 31# L | U/L | (32-103) |
| ALT(GPT) | 29 | U/L | (7-36) |
| AST(GOT) | 29 | U/L | (15-33) |
| LDH | 290 | U/L | (180-380) |
| [CHO,TG,HDL,LDL] | | | |
| CHOLESTEROL | 7.18# | MMOL/L | (4.78-7.50) |
| | | DESIRABLE | LEVEL <5.17 |
| HDL CHOLESTEROL | 1.29# | MMOL/L | (0.72-1.68) |
| TRIGLYCERIDE | 1.14 | MMOL/L | (0.56-2.09) |
| LDL CHOLESTEROL | 5.37 H | MMOL/L | ( -5.17) |
| | | DESIRABLE | LEVEL <3.33 |
| PROSTATE SP AG | 1.0 | UG/L | (0-4.0) |
| ICD | 7.7 | U/L | (2-9) |
| CEA | 2.5# | UG/L | (0.5-3.5) |
| [(T3U,T4,FTI)] | | | |
| T3 RESIN UPTAKE | 106 | % | (77-129) |
| THYROXINE (T4) | 75 | NMOL/L | (59-155) |
| FTI | 7.9 | | (4.6-11.6) |
| ALPHAFOETO PROT | 3.2 | UG/L | (1-10) |

Singapore General Hospital Pte Ltd

Outram Road Singapore 0316. Tel: 2223322

Telex: RS 28847 GENHOS Fax: 2221720

*A Tradition of Caring & Excellence*

*Figure 6. Example of a biochemistry report.*

Singapore General Hospital

Department of Pathology: Telex: RS 20829 PATLAB Fax: 2228924

RUN ON 10/09/91-1456 — HAEMATOLOGY REPORT — PAGE 1 RUN FOR 24/08/91

TAN,HWA — SGH WARD 47 — NRIC # S01335 .

MALE AGE: 33 — CLASS: B1 RM/BED: 11/2 — ID # 10191075680F

2408:HA0622R REQUEST # HA699102 RECV: 24/08/91 1356 COLLN: 24/08/91 UNK

ORDERED: FBC - JR

[FBC - JR]
[HEMOGRAM JR]

| | | | | | |
|---|---|---|---|---|---|
| WBC | 13.4 H X10(9)/L | (4.0-10.0) | RBC | 4.33 L X10(12)/L | (4.5-6.3) |
| HB | 13.3 L G/DL | (14.0-18.0) | HCT | 40.7 % | (38-52) |
| MCV | 94.1 FL | (76-96) | MCH | 30.7 PG | (27-32) |
| MCHC | 32.6 G/DL | (32-36) | RDW | 12.3 % | (10.9-15.7) |
| PLT | 233 X10(9)/L | (140-440) | MPV | 9.4 % | (6.3-10.1) |
| [DIFF CT] | | | | | |
| LYMPH | 15.0 % | (15-41) | MONO' | 11.1 H % | (2-10) |
| GRAN | 73.9 % | (40-75) | | | |

[BLOOD FILM ]

RBC COMMENT RBCS ARE CRENATED

Singapore General Hospital Pte Ltd

Outram Road Singapore 0316. Tel: 2223322

Telex: RS 28847 GENHOS Fax: 2221720

*A Tradition of Caring & Excellence*

*Figure 7. Example of haematology report.*

figure 10). This has improved the turn-around-time for result reporting and reduced the frequent telephone enquiries for the tracing of results.

*Shadowing (or mirroring)*

As the computer is shared by all laboratory disciplines it is absolutely necessary that downtime is kept to the minimum. The system provides this through a hardware facility called the system fault tolerance. The system is configured with one disc drive and a second disc drive, a shadow drive, attached to the first. The second drive automatically and continuously keeps a real-time mirror image copy of the database of the live disc. Should the live disc malfunction, this second disc drive automatically takes over and services the laboratory network at normal throughput. The system runs 24 hours and a complete backup is done every day from the shadow disc. With this feature, operators can continue to use the system during backup. It takes approximately 45 min to transfer 1 Gb of data to the helical tape drive.

*Archiving*

For histopathology, as results are of lifetime significance, it is always kept on-line. However, after 90 days, the descriptive part of the text in the report is removed from the system. For biochemistry, haematology and microbiology, if a patient's file has not been active for 90 days, the results are archived onto another disc. These results can be recalled when required.

*Colour graphics*

The MAGIC System offers colour graphics on the screen display. In addition to the regular tabular types of data presentation, data can be presented in graphical form, for example line graphs, point graphs and bar graphs.

It is believed that graphs will help improve the quality and efficiency of care delivered to the patient, because it can provide a visualization of patterns and relationships for diagnosis and treatment. Progression, regression and subtle changes in values are more easily perceived graphically than from a table of values.

*Windowing*

The use of winows or pop-up screens is another useful feature of the MAGIC system. Windows allow the operator to focus attention to options he or she is unsure about, and helpful instructions are displayed so that input can be continued without having to leave the screen. The facility saves the operator valuable hours in computing time.

Singapore General Hospital

Department of Pathology: Telex: RS 20829 PATLAB Fax: 2220924

DEPARTMENT OF PATHOLOGY

MICROBIOLOGY REPORT

NAME: CHUA,AH — LOCATION: TTSH WARD 52 — NRIC: S00751

SEX/AGE/RACE: F 60 C — CLASS: C — A/C#: I02910201420

DATE REC'D: 03/07/91 — RM/BED: 3 /

SPEC #: 91:DU000559R — SOURCE: URINE — REQ FORM: U25435

ORDERED: UR DIPSLIDE

URINE CULT DIP-SLIDE
=> DOUBTFUL SIGNIFICANT

VIABLE COUNT >100,000 CFU/ML

(1) ESCHERICHIA COLI

(2) KLEBSIELLA SPECIES

| | E.COLI | KLEB SP |
|---|---|---|
| | ST | ST |
| AMPICILLIN | S | R |
| CEPHALEXIN | S | S |
| GENTA | S | S |
| NALI ACID | S | S |
| COTRIMOX | S | S |
| NITROFU | S | S |

Singapore General Hospital Pte Ltd

Outram Road Singapore 0316. Tel: 2223322

Telex: RS 20047 GENHOS Fax: 2221720

A Tradition of Caring & Excellence

*Figure 8. Example of a microbiology report.*

Singapore General Hospital

Department of Pathology: Telex: RS 28829 PATLAB Fax: 2220924

HISTOPATHOLOGY REPORT

BIOPSY No. : 91:PB026798R — HOSPITAL : SGH WARD 54 GYNAE

PATIENT : TEO,GEOK — DEPT : OBST & GYNAE

NRIC : SJ011 — CLASS : B1

RACE/SEX/AGE : C /F /44 — ACCOUNT NO: I0191071396A

HEAD OF UNIT : DR MAURINE

SUBMITTING PHYSICIAN: DR K H — DATE RECEIVED : 30/08/91

TISSUES
A: UTERUS AND CERVIX, CS - LEFT OVARY

GROSS DESCRIPTION

Uterus, cervix & left ovary. Cervix measuring 3.5x3.5x3cm & appears unremarkable. the uterus or partially bisected end measuring 9.5x8x5.5cm. Endometrium is thin. The post uterine wall shows a diffuse whorled white area about 3.5x5x4cm. Left fallopian tube measures 6cm & ovary measuring 2.5x2x1.3cm. (Cervix-2,endometrium-1,whorled white area-2,tube-1,ovary-2). (DR. CHEW).

MICROSCOPIC DESCRIPTION

Cervix : Shows chronic cervicitis with squamous metaplasia.

Endometrium : Shows basal endometrium with focal pseudodecidualised stroma.

Myometrium : Shows islands of endometrial glands and stroma interspersed amongst hypertrophic smooth muscle fibre bundles. Islands of endometrial glands and stroma are also seen in the ragged area on the surface of the posterior uterine wall.

The left fallopian tube is unremarkable.

The left ovary shows corpora albicantes, a few of which have central cystic spaces.

No definite endometrial cysts are seen.

DIAGNOSIS

THLSO Specimen : Adenomyosis.

Pathologist :

(DR.)
T82900: M76510 <ANI>

10/09/91
PAGE 1 (END)

Singapore General Hospital Pte Ltd

Outram Road Singapore 0316. Tel: 2223322

Telex: RS 20047 GENHOS Fax: 2221720

A Tradition of Caring & Excellence

*Figure 9. Example of a histopathology report.*

### *Quality-control monitoring routine*

In addition to the usual quality-control (QC) routines of daily QC logs, summaries of QC specimen values over selected time frames, Levy-Jenning plots, there is also a QC routine called the 'Multi-Rule Quality Control Routine'. With this routine, QC specimens are given user defined pre-set limits (for instance mean ± 2 × S.D.). Each time such a QC specimen is run with a batch of patients' specimens, the QC test value can be viewed either on-line through the automated instrument on-line viewing mode or through worksheet entering procedures to verify the validity of the batch results. This feature allows real-time on-line quality control.

### *Automated instrument downloading*

The automated instrument routine allows capture of results on-line as they emerge from the automated instruments. Both haematology and biochemistry have all their fully automated instruments linked on-line to the computer. These instruments handle approximately 80% of the workload of these laboratories. The speed of acquisition and processing of data has been greatly increased, and errors caused by manual transcription of large volumes of laboratory data have been eliminated with the direct link of the MAGIC System to the host IBM. More than 80% of the reports for haematology and biochemistry are printed directly in the wards within 8 h, the majority within 4 h of test requests.

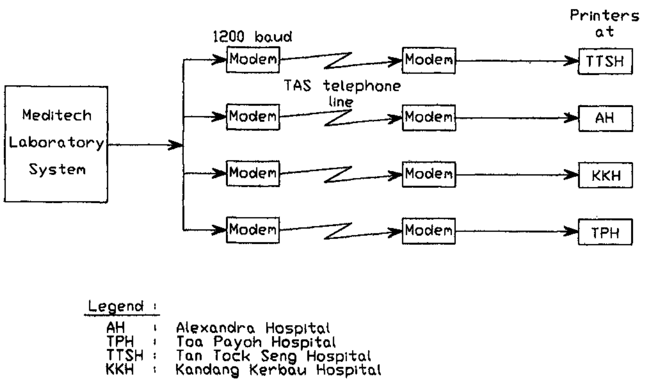

*Figure 10. Transmission of laboratory reports to four other major hospitals using modems, telephone lines and on-site printing terminals.*

A special feature called the instrument download routine allows the operator to send specimen and test requests directly to an instrument which has a two-way communication with the computer. The user may download data either for individual specimens or for an entire batch. This feature allows automatching of results to the correct specimen for immediate transmission.

### Discussion

Several important factors have contributed to the successful implementation of the on-line laboratory computer. Prior experience of the staff of biochemistry and haematology was an advantage as they had become familiar with the concept and use of a system for data processing: this experience reduced the time needed for learning to live with the new system.

A familiarization progamme was offered to staff of the sections that were being introduced to the laboratory computer for the first time. Since staff interest, motivation and co-operation is essential for any new system implementation, great care was taken to ensure that all members of staff were kept fully briefed as the implementation programme developed. Staff co-operation, in terms of suggestions and criticism, was actively encouraged and this approach generated a great deal of enthusiasm and resulted in ready acceptance.

The ease of hardware operation, the user-friendliness of the various application programs and the good performance of the system were important in the gaining of confidence and acceptance of the laboratory staff.

All programs produced information and instructions in simple English on screen; in addition, requests for direction are displayed whenever more than one option occurs during a run.

The MAGIC System described has significantly improved laboratory mangement and quality of work and has resulted in enhanced efficiency and productivity.

### References

1. Tan, I. K., Tay, B. S., Lim, S. H., Taw, C. K. and Ng, M. C., Electronic-data-processing in the clinical biochemistry laboratories in Singapore. In Proceedings of the 9th Malaysia–Singapore Congress of Medicine (1974).
2. Tan, I. K., Tay, B. S., Lim, S. H., Taw, C. K. and Ng, M. C., *Singapore Medical Journal*, **16** (1975), 166.
3. Tan, I. K., *Israel Journal of Medical Science*, **13** (1977), 20.
4. Tan, I. K. and Jacob, E., *Annals of the Academy of Medicine Singapore*, **11** (1982), 424.
5. Tan, I. K., Jacob, E. and Lim, S. H., Rubin, M. (Ed.) *Computerization and Automation in Health Facilities* (CRC Press, Florida, 1984), 111.
6. Abson, J., Prall, A. and Wootton, I. D. P., *Annals of Clinical Biochemistry*, **14** (1977), 307.
7. Abson, J., Prall, A. and Wootton, I. D. P., *Annals of Clinical Biochemistry*, **14** (1977), 315.
8. Abson, J., Prall, A. and Wootton, I. D. P., *Annals of Clinical Biochemistry*, **14** (1977), 323.
9. Undrill, P. E. and Gibson, P., *Annals of Clinical Biochemistry*, **15** (1978), 235.
10. Jomain, P. A. and Owen, J. A., *Annals of Clinical Biochemistry*, **15** (1978), 276.
11. Morgan, L. M., McConnell, G., Chandler, E. and Welshman, S. G., *Annals of Clinical Biochemistry*, **17** (1980), 47.
12. Rappoport, A. E., In Proceedings of the XI International Congress of Clinical Chemistry (Vienna, 1982), 1357.
13. Roesler-Englhardt, A., In Proceedings of the XI International Congress of Clinical Chemistry (Vienna, 1982), 1363.
14. Jarosch, E., In Proceedings of the XI International Congress of Clinical Chemistry (Vienna, 1982), 1431.
15. Hohenwallner, W., Wimmer, E. and Sommer, R., In Proceedings of the XI International Congress of Clinical Chemistry (Vienna, 1982), 1437.
16. Niederer, R., Flury, R. and Keller, H., In Proceedings of the XI International Congress of Clinical Chemistry (Vienna, 1982), 1443.
17. Flynn, F. V., In Albert K. G. M. M. (Ed.), *Recent Advances in Clinical Biochemistry* (1978), 255.
8. Tan, I. K., In Yu, M. (Ed.), Annual Report of the Department of Pathology (Ministry of Health Singapore, 1983), 20.

